# Uncovering Molecular Heterogeneity in the Kidney With Spatially Targeted Mass Spectrometry

**DOI:** 10.3389/fphys.2022.837773

**Published:** 2022-02-11

**Authors:** Angela R. S. Kruse, Jeffrey M. Spraggins

**Affiliations:** ^1^Department of Biochemistry, Vanderbilt University, Nashville, TN, United States; ^2^Mass Spectrometry Research Center, Vanderbilt University, Nashville, TN, United States; ^3^Department of Cell and Developmental Biology, Vanderbilt University, Nashville, TN, United States; ^4^Department of Chemistry, Vanderbilt University, Nashville, TN, United States

**Keywords:** mass spectrometry, kidney, proteomics, metabolomics, lipidomics, multimodal imaging, HuBMAP, KPMP

## Abstract

The kidney functions through the coordination of approximately one million multifunctional nephrons in 3-dimensional space. Molecular understanding of the kidney has relied on transcriptomic, proteomic, and metabolomic analyses of kidney homogenate, but these approaches do not resolve cellular identity and spatial context. Mass spectrometry analysis of isolated cells retains cellular identity but not information regarding its cellular neighborhood and extracellular matrix. Spatially targeted mass spectrometry is uniquely suited to molecularly characterize kidney tissue while retaining *in situ* cellular context. This review summarizes advances in methodology and technology for spatially targeted mass spectrometry analysis of kidney tissue. Profiling technologies such as laser capture microdissection (LCM) coupled to liquid chromatography tandem mass spectrometry provide deep molecular coverage of specific tissue regions, while imaging technologies such as matrix assisted laser desorption/ionization imaging mass spectrometry (MALDI IMS) molecularly profile regularly spaced tissue regions with greater spatial resolution. These technologies individually have furthered our understanding of heterogeneity in nephron regions such as glomeruli and proximal tubules, and their combination is expected to profoundly expand our knowledge of the kidney in health and disease.

## Introduction

The kidney is a complex and vital organ that filters waste products from the blood, stabilizes electrolyte and water content, and secretes essential hormones (Tryggvason and Wartiovaara, 2005; Ferraro and Fuster, 2021). It functions through nuanced coordination of approximately one million nephrons in 3-dimensional space. Nephrons can be further sub-divided into functional tissue units (FTUs) including vasculature, ducts, tubules, and glomeruli, each with unique molecular functions. FTUs are influenced by proximity to other structures and location within the organ. Individual glomeruli and tubules vary in vascular architecture, molecular environment and drug distributions (Kang et al., 2005; Postnov et al., 2015; Kafarov et al., 2020). This heterogeneity is especially important in the context of renal disease, which can uniquely impact individual FTUs (Weening et al., 2004; Fogo, 2015; Aguayo-Mazzucato et al., 2017). Traditionally, our molecular understanding of renal disease comes from global transcriptomic, proteomic, and metabolomic analyses of kidney lysates. These bulk analyses offer deep and comprehensive molecular coverage and are invaluable for sample profiling and disease biomarker identification (Mayer et al., 2012). However, cell identity and spatial context are lost when tissues are homogenized, and molecular changes at the cellular or FTU level are diluted in bulk tissue analyses. This can result in the lack of detection of inter-individual and disease-associated variation, as well as inability to identify rare cell populations. Molecular characterization of dissociated cells provides cellular information lacking in bulk analyses but does not retain spatial context (Koehler et al., 2020). In addition, enzymatic dissociation of tissues can disrupt the cellular environment and preclude analysis of extracellular matrix molecules that can be relevant in fibrotic kidney disease (Autengruber et al., 2012). Recently, spatially targeted mass spectrometry (MS) technologies have emerged that provide a deeper understanding of the role localized cell types, cellular neighborhoods, and FTUs play in underlying pathomechanisms (Autengruber et al., 2012; Ryan et al., 2019). Each of these MS technologies has unique benefits and drawbacks for the study of human organs. This review highlights the application and potential of spatially targeted MS to illuminate the underlying molecular drivers of kidney health and disease.

## Spatial Mass Spectrometry Technologies

**FIGURE 1:**
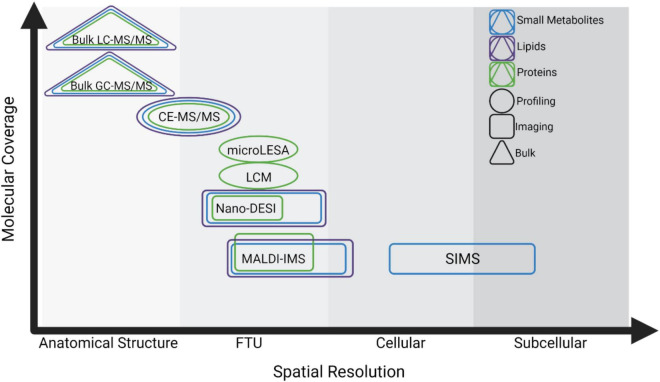
Summary of spatial MS technologies and the trade-offs between spatial resolution and molecular coverage. Technologies are characterized based on their spatial resolution from the level of anatomical structure (>500 μm), Functional Tissue Unit (FTU, ∼50–500 μm), cellular (∼10–50 μm), and subcellular (<10 μm). Triangles indicate technologies for analysis of bulk or homogenate tissues, circles indicate profiling experiments, and rectangles indicate imaging experiments. Methods for analyzing small metabolites, lipids, and proteins are shown in blue, purple, and green, respectively.

Spatially targeted MS technologies are characterized as either profiling experiments, where a single spectral signature is collected from a discrete cell type or FTU, or as imaging experiments where MS data are collected from an array of measurement locations (i.e., pixels) to visualize molecular distributions *in situ* (Figure 1). Micro-liquid extraction surface analysis (microLESA) is a profiling approach using a robotic fluidic printer to deposit trypsin droplets to specific tissue regions for surface protein digestion (Ryan et al., 2019; Guiberson et al., 2021). Peptides are then recovered using a larger droplet and subjected to liquid chromatography-tandem MS (LC-MS/MS) for protein identification. Laser capture microdissection (LCM) is also commonly employed in profiling experiments and involves dissection of specific sample regions using a cutting laser and subsequent collection into a sample tube using laser propulsion. Collected regions can be analyzed individually or pooled for protein, lipid, or small metabolite profiling (Datta et al., 2015; Knittelfelder et al., 2018; Sigdel et al., 2020). Although the achievable spatial resolution is limited, LCM can also be integrated into quasi-imaging workflows by dissecting tissue in a grid pattern in which each collected square becomes a voxel (Piehowski et al., 2020). Each region can be subjected to proteomic analysis using methods specialized for low sample input such as nanodroplet processing in one pot for trace samples (NanoPOTS), and voxels can be reconstructed to show intensity variation throughout the sample (Zhu et al., 2018; Piehowski et al., 2020).

Imaging mass spectrometry (IMS) is a powerful technology to construct spatial maps of analytes without labeling and in an untargeted manner (Caprioli et al., 1997; Gode and Volmer, 2013; Norris and Caprioli, 2013; Wu et al., 2013; Nilsson et al., 2015; Spengler, 2015). The most common IMS methods use soft ionization such as matrix-assisted laser desorption (MALDI) and desorption electrospray ionization (DESI) (Roach et al., 2010; Eberlin et al., 2011). In MALDI IMS workflows, tissue samples are coated with a matrix that assists with desorption and ionization of endogenous analytes (Franz and Michael, 2000). The tissue surface is then ablated using a laser in a raster pattern, where each laser spot produces a spectrum detecting hundreds to thousands of ions (Caprioli et al., 1997; Norris and Caprioli, 2013; Spraggins et al., 2019; Martín-Saiz et al., 2021). Spectral information from each laser spot (i.e., pixel) is reconstructed to show relative analyte intensity and distribution throughout the sample (Caprioli et al., 1997; Norris and Caprioli, 2013; Spraggins et al., 2019; Martín-Saiz et al., 2021). DESI and nano-DESI workflows use ambient liquid extraction of small tissue regions at regularly spaced measurement regions followed by introduction to a mass spectrometer inlet or primary capillary for electrospray ionization (Roach et al., 2010; Eberlin et al., 2011). MS data from sampled tissue coordinates can similarly be reconstructed into spatial maps in DESI and nano-DESI IMS workflows. Secondary-ion mass spectrometry (SIMS) has achieved the highest spatial resolution to date, but the high energy required for ionization limits the size of molecule that can be analyzed, making this technique more widely applied for analysis of elements and smaller biomolecules (<1,000 Da) rather than larger lipids, peptides, and proteins (Wu and Odom, 1996; Heeren et al., 2006; McDonnell et al., 2006).

Each of these strategies requires a trade-off between spatial resolution and sensitivity, where methods approaching cellular or subcellular resolution often detect fewer analytes. Sample preparation and ionization methods also impact the molecular class that can be analyzed. This continuum is especially notable in spatially targeted MS, and researchers must use their judgment to select the technology most suited to their experimental goals (Figure 1).

## Proteomics

Proteomics offers direct information about downstream effects of transcriptional and translational regulation on cellular function and does not require extrapolation from transcript data (Gry et al., 2009). MS-based proteomics provides an advantage over antibody-based techniques in that it is untargeted, highly multiplexed, and requires no *a priori* knowledge of antibody targets. It also retains information about truncated and post-translationally modified proteoforms that can be impacted by renal disease (Yassine et al., 2016). Spatial proteomics is uniquely advantageous since it can specifically assess protein regulation in individual kidney FTUs and cell types, and has been used to show that adjacent nephrons vary at the proteomic level (Höhne et al., 2018). Both profiling and imaging MS approaches have been applied to the study of the kidney.

Spatial proteomics profiling experiments rely on the ability to analyze increasingly small amounts of starting material, requiring advances in sample preparation, chromatography, and instrumentation. Protocols employing filters, magnetic beads, or micro-volumes minimize sample loss by performing enzymatic digestion in one tube or droplet (Hughes et al., 2014; Kulak et al., 2014; Moggridge et al., 2018; Xu et al., 2019). NanoPOTS and MicroPOTS have facilitated near-single cell proteomics and are designed for low-input samples (Xu et al., 2019). Polished sample tubes and mass-spectrometry compatible detergents additionally minimize sample loss and the need for detergent removal (Norris et al., 2003, 2005; Grzeskowiak et al., 2016). Ultra-low flow chromatography and fractionation, and capillary electrophoresis can improve protein separation and address the wide range of protein concentrations found in biological samples (Waanders et al., 2008; Aebersold and Mann, 2016; Greguš et al., 2020; Kelly, 2020; Xiang et al., 2020). Pairing these sample preparation and separation techniques with high-resolution MS instrumentation can further facilitate low-input proteomics analysis (Norris et al., 2005). In addition, nanopore sequencing can now be used for single-cell proteomics and will likely be integrated into low-input proteomics workflows (Brinkerhoff et al., 2021).

These advances enabled multiple studies to characterize renal FTU proteomes. The combination of LCM with low-loss sample preparation and chromatography for LC-MS/MS proteomics has been especially successful for analysis of kidney FTUs. Thousands of proteins can be identified from single human or murine glomeruli or 30–40 single microdissected cells (Waanders et al., 2009; Sigdel et al., 2020). One study identified 67 proteins only detected in glomeruli and 25 unique to proximal tubules, with many additional proteins shared by both regions being conserved housekeeping and cytoskeletal proteins (Sigdel et al., 2020). Notably, this study found that proximal tubule proteins comprised a greater fraction of the homogenate proteome than glomerular proteins, and known glomerular markers such as podocin, eva-1 homolog B, and claudin-5 could be identified in dissected glomeruli but not in kidney homogenate (Guiberson et al., 2021). This underscores the value of a spatially targeted approach to study glomeruli (Sigdel et al., 2020). Another LCM-based study investigated proteomic changes associated with proteinuric kidney disease in glomeruli and tubules from murine and human samples. This work implicated a suite of proteins including lysosomal-associated membrane protein 1, cathepsin proteases, albumin, and extracellular matrix proteins in proteinuric kidney disease and proposed further research into cathepsins as potential therapeutic targets (Höhne et al., 2018). Analyses of single glomeruli and glomerular extracellular matrix consistently identify cathepsin proteases and proteins associated with vesicular transport and cellular component organization as differentially abundant in diseased kidney tissue (Hobeika et al., 2017; Höhne et al., 2018; Sigdel et al., 2020). Proteins enriched in proximal tubules were consistently involved in solute transport and small molecule metabolic processing, offering the intriguing possibility of measuring corresponding differences in metabolite abundance and localization (Hobeika et al., 2017; Höhne et al., 2018). Taken together, spatially targeted proteomics of kidney FTUs are invaluable to understanding renal FTU heterogeneity.

Imaging mass spectrometry is a powerful and complementary technology to spatially map proteins and peptides in tissue sections in an untargeted manner and with greater proteomic coverage than antibody-based imaging (Caprioli et al., 1997). Protein imaging requires minimal sample preparation and can be used to visualize proteins under ∼60 kDa depending on MS instrumentation (Chaurand et al., 2006; Norris and Caprioli, 2013). Although its spatial resolution is far superior to profiling-based technologies, a large proportion of the proteome is not available for analysis by this technique. In contrast, peptide imaging provides better proteomic coverage but requires more sample preparation and can suffer from delocalization during on-tissue enzymatic digestion of endogenous proteins (Judd et al., 2019). A major challenge for protein and peptide IMS is ion identification. Most protein IMS experiments rely on exact mass matching within a certain ppm error for identification. Recent computational tools allow for high throughput matching of *m/z* values with candidate identifications based upon intact mass and spatial correlation (Guo et al., 2021). Thus, advances in sample preparation, instrumentation, and computation are improving the feasibility and interpretation of protein and peptide IMS.

Imaging mass spectrometry has been applied to image kidney proteins and peptides, and has great potential as a tool for biomarker discovery and disease characterization (Caprioli et al., 1997; Lalowski et al., 2013; Jones et al., 2014; Casadonte et al., 2015; Srinivasu et al., 2021). Protein IMS was used to identify accumulated cortical transthyretin as a protein biomarker for gentamicin-induced kidney toxicity, and to spatially characterize angiotensin metabolism in murine kidneys (Meistermann et al., 2006; Grobe et al., 2012). Peptide IMS was used to determine that amyloid P component, apolipoprotein E, and vitronectin co-localize with renal amyloid deposits in human biopsy samples (Casadonte et al., 2015). Yet another study found differences in localization of α-enolase peptides in rat kidneys after treatment with nanoparticles commonly found in cosmetic and medical products (Srinivasu et al., 2021). Peptide IMS signal can be enhanced through secondary ionization (MALDI-2) and has been used to show localization of hemoglobin subunit proteins, glutathione-S-transferase, and pyruvate kinase to glomeruli, cortex, and medulla, respectively (McMillen et al., 2021). These IMS studies have benefited from the ability to visualize changes in analyte localization in broad tissue areas, and have leveraged microextraction or homogenate analyses with deeper proteomic coverage to confirm protein identifications (Grobe et al., 2012).

## Small Molecule Metabolomics

Mass spectrometry-based metabolomics is essential in basic and clinical renal research (Abbiss et al., 2019). Here, metabolites are defined as small (<1,000 Da) molecules such as amino acids, nucleotides, mono- and disaccharides, and steroids that can be hydrophilic, hydrophobic, or amphipathic (Bijlsma et al., 2006). Liquid or gas chromatography-based metabolomics are routinely used to assess aminoaciduria in clinical samples or tissue homogenates (Rhee, 2018; Abbiss et al., 2019). Early work on the kidney profiled patient samples for disease biomarkers and resulted in the clinical tests now available to physicians (Cisek et al., 2016; Luft, 2021). However, general metabolic markers do not provide information about inter-nephron variation, and there is a gap in understanding sources of metabolic dysfunction on a spatial level and relating these to specific proteins. For example, amino acid transporters have been found to differ in proximal tubules within the same tissue section, implying that solute transport may be performed differently among nephrons and may be contributing uniquely to aminoaciduria and other kidney dysfunctions (Höhne et al., 2018).

Imaging mass spectrometry is uniquely powerful for kidney metabolomics because it is one of few methods that can spatially map metabolites within tissue, since these molecules are not amenable to antibody-based visualization (Prentice et al., 2017). Metabolite IMS has been used to characterize drug distribution in murine kidneys (Römpp et al., 2011), adenosine triphosphate and monophosphate in diabetic murine kidneys (Miyamoto et al., 2016), N-linked glycans in murine kidney (Gustafsson et al., 2015), and is extensively reviewed in Prentice et al. (2017). Metabolites can be routinely imaged with pixel sizes as small as 10 μm and their detection can be enhanced by gas-phase separation approaches such as trapped ion mobility separation (TIMS) (Djambazova et al., 2020; Neumann et al., 2020). Small metabolite IMS in human kidney samples was performed at a spatial resolution of 20 μm and allowed for the detection of >200 unique species using a timsTOF mass spectrometer in qTOF mode only (i.e., without TIMS activated) and >350 species after applying TIMS (Neumann et al., 2020). This study revealed unique distributions of metabolites including argininic acid, acetylcarnitine, and choline in the cortex, medulla, and renal pelvis, respectively (Neumann et al., 2020). Nano-DESI IMS was similarly used to show localization of propionylcarnitine, methylhistidine, sorbitol to the cortex, outer medulla, and inner medulla, respectively (Bergman et al., 2019). Acylcarnitine was shown to accumulate in the cortex of early-diabetic mice (Bergman et al., 2019). These approaches illustrate the excellent spatial resolution achievable by metabolite IMS and provide robust methods to visualize these molecules that cannot routinely be imaged using antibodies or affinity reagents. Future work could integrate metabolomic analyses of isolated FTUs with IMS to leverage the molecular coverage of the former with the spatial resolution of the latter.

## Lipidomics

Lipids play crucial and diverse roles in the kidney from establishment of cellular structure and stability to cell-cell interactions (Kinnunen et al., 2012; Balla, 2013). Lipids are metabolized in the kidney via receptor-mediated uptake of plasma lipids in proximal tubules (Moestrup and Nielsen, 2005). Chronic renal disease is associated with abnormal lipid metabolism, elevated apolipoprotein abundance, and elevated plasma lipid levels (Trevisan et al., 2006). Oxidative stress and insulin resistance have been implicated in lipid-mediated renal damage, but the underlying genetic, proteomic, and metabolomic mechanisms are not understood (Trevisan et al., 2006). Additionally, altered renal lipid distribution has been associated with nephron dysfunction resulting from pathogen infection (Perry et al., 2019), polycystic kidney disease (Ruh et al., 2013), early diabetes and obesity (Miyamoto et al., 2016; Sugimoto et al., 2016; Bergman et al., 2019), and kidney injury (Rao et al., 2016). MS-based lipidomics globally characterizes how lipid class and the molecular structure influence these processes, and is uniquely informative in the context of renal disease.

Lipid IMS has been widely applied to the kidney and is further reviewed in Miyamoto et al. (2016). Gangliosides, sulfoglycosphingolipids, lysophospholipids, and phosphatidylethanolamines, sphingolipids, and lysolipids were shown to accumulate and spatially relocalize in the kidney due to diabetic nephropathy and severe ischemic injury in murine and porcine samples (Grove et al., 2014; Miyamoto et al., 2016; van Smaalen et al., 2019). Two ether-linked phospholipids were implicated as biomarkers for acute kidney injury in a murine model using sequential window acquisition of all theoretical spectra (SWATH) lipidomics and IMS (Rao et al., 2016). These phospholipids were shown to accumulate in proximal tubules, supporting the combination of profiling and imaging MS to characterize lipid abundance and localization (Rao et al., 2016). Another study showed that the ganglioside NeuGc-GM3, but not other ganglioside species, and several lysophospholipids accumulated in glomeruli of diabetic mice, while long-chain sulfoglycolipids accumulated in renal tubules of diabetic mice (Grove et al., 2014). Amadori-modified phosphatidylethanolamines were also detected in the renal cortex of diabetic mice, providing insight into the metabolic impacts of diabetes (Grove et al., 2014). Each of these approaches showed profound redistribution of lipid species in response to renal disease. To provide further insight into the lipidome and disease, technologies are linking specific lipid species with kidney FTUs based upon histology-informed segmentation of lipid IMS data (Martín-Saiz et al., 2021). These studies illustrate the utility of IMS to detect global lipidomic changes in disease and implicate diverse lipid classes in normal kidney function and pathogenesis.

## Conclusion and Perspective

Technological advancement fundamentally changes the scale and strategy of scientific research. High-performance mass spectrometry and spatial technologies have moved us into an era of “big data” where the amount of molecular information collected from a single sample would have been previously inconceivable (Beckmann and Lew, 2016). To move beyond simply collecting big data to comprehensive interpretation of complex datasets, we assert that we are beginning an era focused on “multimodal data integration.” Scientists will need to cooperatively link and automatically mine large datasets to understand intricate networks of cellular and molecular interactions across vast spatial scales (e.g., anatomical regions to single cells) and wide-ranging molecular classes (e.g., RNA, proteins, lipids, and metabolites). Multi-institutional consortia are working to address this challenge by constructing molecular atlases of human cells and organs that integrate imaging and omics technologies using spatial anchors through common coordinate frameworks and/or anatomical links (Abbasi, 2017; Hu, 2019; Rozenblatt-Rosen et al., 2020; El-Achkar et al., 2021). These consortia are prioritizing the establishment of broadly accepted standards and quality control for data collection, precise recording of biopsies and tissue sections locations within intact organs, and recording thorough donor metadata data in accessible and stable repositories (Lynch, 2008; Hu, 2019; El-Achkar et al., 2021).

**FIGURE 2:**
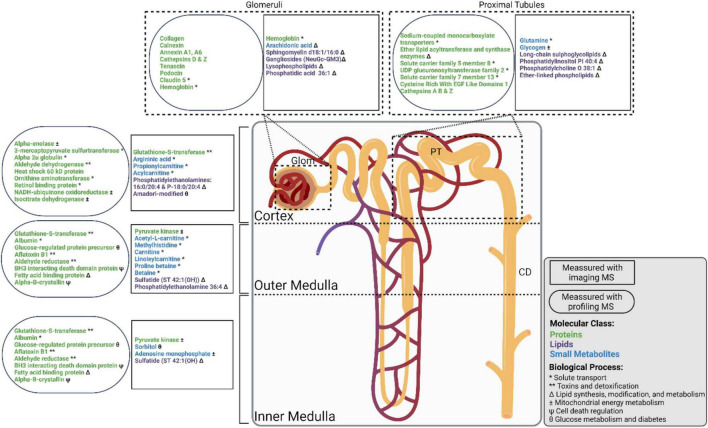
Nephron showing select molecular groups observed in the cortex, outer medulla, inner medulla, glomeruli (Glom), proximal tubules (PT), and collecting ducts (CD). Molecular observations made using imaging mass spectrometry (MS) and profiling MS experiments are shown in rectangles and ovals, respectively. Proteins, small metabolites, and lipids, are shown in green, blue, and purple text, respectively. Biological processes are indicated as follows: * Solute transport, ** Toxins and detoxification, Δ Lipid synthesis, modification, and metabolism, ± Mitochondrial energy metabolism, Ψ Cell death regulation, θ Glucose metabolism and diabetes.

These large-scale kidney research projects are balancing the application of established multi-omic technologies with continued development of cutting-edge spatially targeted MS approaches. Spatially targeted proteomics utilizing profiling strategies has been more widely applied to the kidney than other technologies, and therefore individual glomerular and tubule proteomes are more well characterized (Betsholtz et al., 2007; Höhne et al., 2018; Hoyer et al., 2019; Späth et al., 2019; Koehler et al., 2020; Sigdel et al., 2020; Banki et al., 2021). Metabolomics has been applied to patient samples and kidney homogenates to great effect, and IMS has broadly characterized the spatial distribution of select small metabolites (Abbiss et al., 2019; Neumann et al., 2020; Zhang et al., 2020). Similarly, lipidomics has been used to characterize kidney homogenates, and IMS has generated spatial lipid maps (Rao et al., 2016; Lukowski et al., 2020; Zhang et al., 2020; Martín-Saiz et al., 2021). The next challenge will be to integrate these analytical modalities into workflows combining multiple spatially targeted MS technologies and to develop tools necessary to perform these analyses at scale across statistically relevant numbers of samples. The integration of these modalities in a systems-biology approach can provide us with a more comprehensive understanding of kidney biology (Figure 2; Mayer et al., 2012; Cisek et al., 2016; Rhee, 2018; Zhang et al., 2018; Neumann et al., 2021a).

High performance computing and development of necessary machine learning algorithms are playing an important role in technology integration. In addition to combining spatially targeted MS approaches, we anticipate that it will become more common for these data to be combined with other advanced molecular imaging technologies such as microscopy and spatial transcriptomics. Examples of this have already demonstrated integration of spatially targeted MS data with autofluorescence microscopy and multiplexed immunohistochemistry approaches such as imaging mass cytometry (IMC) and co-detection by indexing (CODEX) to molecularly characterize and discover markers for kidney FTUs and cell types (Patterson et al., 2018; Singh et al., 2019; Martín-Saiz et al., 2021; Neumann et al., 2021a,b). To enable these multimodal approaches, computational tools are emerging that automatically annotate, integrate, and mine molecular imaging data from orthogonal technologies for unbiased data interpretation and identification of candidate biomarkers (Van de Plas et al., 2015; Palmer et al., 2017; Vollnhals et al., 2017; Balluff et al., 2019; Race et al., 2020; Martín-Saiz et al., 2021; Tideman et al., 2021).

In summary, spatially targeted MS is a powerful set of technologies for the discovery of molecular profiles of critical FTUs and cell types in the kidney. As the field matures, multimodal data integration will certainly become more common requiring interdisciplinary, and often multi-institutional collaborations bringing together researchers with a wide array of expertise including cell biologists, pathologists, analytical chemists, computer scientists, and mathematical engineers. The application of this diverse set of expertise and technological capabilities is expected to dramatically enhance our understanding of the cellular and molecular makeup of the kidney to personalize medical care and improve health outcomes.

## Author Contributions

AK and JS completed the original manuscript draft and contributed to critical review and editing of the manuscript. Both authors contributed to the article and approved the submitted version.

## Conflict of Interest

The authors declare that the research was conducted in the absence of any commercial or financial relationships that could be construed as a potential conflict of interest.

## Publisher’s Note

All claims expressed in this article are solely those of the authors and do not necessarily represent those of their affiliated organizations, or those of the publisher, the editors and the reviewers. Any product that may be evaluated in this article, or claim that may be made by its manufacturer, is not guaranteed or endorsed by the publisher.

## Abbreviations

MS: mass spectrometry
FTU: functional tissue unit
PT: proximal tubule
Glom: glomerular
CD: collecting duct
microPOTS: microliter processing in one pot for trace samples
LCM: laser capture microdissection
microLESA: micro, liquid extraction surface analysis
MALDI: matrix assisted laser desorption/ionization
DESI: desorption electrospray ionization
IMS: imaging mass spectrometry.
